# Macular Rod Function in Retinitis Pigmentosa Measured With Scotopic Microperimetry

**DOI:** 10.1167/tvst.10.11.3

**Published:** 2021-09-02

**Authors:** Arun K. Krishnan, Alejandro J. Roman, Malgorzata Swider, Samuel G. Jacobson, Artur V. Cideciyan

**Affiliations:** 1 Scheie Eye Institute, Perelman School of Medicine, University of Pennsylvania, Philadelphia, PA, USA

**Keywords:** microperimetry, rod vision, automated perimetry

## Abstract

**Purpose:**

To investigate the validity and reliability of macular rod photoreceptor function measurement with a microperimeter.

**Methods:**

Macular sensitivity in dark-adapted retinitis pigmentosa (RP) patients (22 eyes; 9–67 years of age) and controls (five eyes; 22–55 years of age) was assessed with a modified Humphrey field analyzer (mHFA), as well as a scotopic microperimeter (Nidek MP-1S). Sensitivity loss (SL) was estimated at rod-mediated locations. All RP eyes were re-evaluated at a second visit 6 months later. The dynamic range of the MP-1S was expanded with a range of neutral-density filters (NDFs).

**Results:**

In controls, a 4 NDF was used at all macular locations tested. In patients with RP, 0 to 3 NDFs were used, depending on the local disease severity. At rod-mediated locations (*n* = 281), SL estimates obtained with the MP-1S were highly correlated (*r* = 0.80) with those of the mHFA. The inter-perimeter difference of SL averaged less than 3 decibels (dB) with all NDFs, except those with most severe locations evaluated with a 0 NDF, where the difference averaged more than 6 dB. The results were similar on the second visit.

**Conclusions:**

The MP-1S estimates of SL are highly correlated with those of the mHFA over a wide range of disease severity replicated at two visits; however, there was an unexplained bias in the magnitude of SL estimated by the MP-1S especially at loci with severe disease.

**Translational Relevance:**

MP-1S scotopic microperimetry can be used to evaluate changes to macular rod function, but evaluation of treatment potential by quantitative comparison of SL to retinal structure will be more challenging.

## Introduction

Mutations in >300 genes cause inherited retinal diseases (IRDs), due to dysfunction and/or degeneration of photoreceptors.^1^^,^^2^ Retinitis pigmentosa (RP) is one of the more common clinical diagnoses associated with IRDs. The great majority of IRDs affect rod and cone photoreceptors to different degrees. The photoreceptor identity and disease severity vary not only with molecular defect but also with retinal location. In terms of understanding the phenotype, stage of disease severity, natural history, and response to treatments, it is important to measure the spatial distribution of rod and cone function and any changes over time.

Assessment of the retinal distribution of cone function is relatively easy, as it can be performed with light-adapted automated perimetry.^3^ In most but not all cases,^4^^–^^6^ use of light adaptation desensitizes rod function^7^^,^^8^ and allows measurement of cone function. On the other hand, estimating rod function in IRDs can be more challenging despite the rod photoreceptors being the dominant cell type across most of the normal human retina. In dark-adapted normal eyes, light sensitivity is dominated by the rods across the retina except at the rod-free foveola. In dark-adapted IRD eyes, the photoreceptor source of sensitivity at any retinal location is not known a priori and depends on the distribution of disease. However, use of blue stimuli near the peak of rhodopsin absorption tends to shift the balance toward rod function due to the Purkinje shift.^9^

Retinal distribution of sensitivity under dark-adapted conditions can be performed with static computerized perimetry under free-viewing conditions whereby stable and foveal fixation to a stationary target by the subject defines the localization of light stimuli being presented. However, patients with IRDs with foveal disease can have extrafoveal and/or unstable fixation, which makes it difficult to reliably associate visual sensitivity to a specific retinal location with a free-viewing perimeter. Use of retina-tracking perimetry, also known as microperimetry, allows evaluation of specific locations in the central retina independent of the subject's ability to fixate.^10^ Additional difficulties may arise from measurements at abrupt health–disease transitions in the retina, even in stably fixating patients. Standard versions of the commercial microperimeter tend to have a dim background and can provide estimates of sensitivity resulting from rod or cone function depending on the local disease stage.^10^ Here we evaluated the validity and reliability of a scotopic microperimeter^11^^–^^18^ to obtain macular rod function with blue stimuli in RP eyes. We specifically chose to include patients with stably fixating eyes so that free-viewing and retina-tracking results can be fairly compared.

## Methods

### Human Subjects

Eleven patients (ages, 9–67 years; 22 eyes) with RP and five control subjects (ages, 22–55 years; five eyes) participated in this research. In addition to the standard clinical examination, RP subjects were evaluated with two perimeters at each visit for two visits, separated by 6 months. All visual field testing was done after dilation of pupils (using 1% tropicamide and 2.5% phenylephrine) in fully dark-adapted (at least 45 minutes) eyes. All subjects with RP demonstrated central fixation, and the best-corrected visual acuity ranged from 20/16 to 20/32 Snellen equivalent. Demographics of the subjects, including refraction, acuities, lenses, and macular status, is provided (Table). The tenets of the Declaration of Helsinki were followed, and informed consent, assent, and parental permission were obtained. The research was approved by the institutional review board at the University of Pennsylvania.

**Table.** Clinical Characteristics of Subjects With RP

		Manifest Refraction		BCVA	
Patient ID	Age^a^/Gender	Right Eye	Left Eye	Right Eye	Left Eye
P1	56/F	–0.25 DS/−0.75 DC × 22	–1.75 DS/–0.25 DC × 24	20/16	20/20
P2	22/F	Plano	Plano	20/16	20/16
P3	29/M	–4.50 DS/−0.50 DC × 140	–5.00 DS/–0.75 DC × 31	20/16	20/16
P4	59/M	–0.25 DS/–0.50 DC × 135	–0.75 DS	20/16	20/20
P5	43/F	–1.25 DS/−1.25 DC × 66	–1.50 DS/–1.00 DC × 93	20/16	20/20
P6	44/F	–2.25 DS/−2.25 DC × 20	–2.00 DS/–1.50 DC × 169	20/32^b^	20/25^b^
P7	53/M	+0.25 DS/−1.00 DC × 72	Plano	20/20^c^	20/20^c^
P8	9/F	–0.75 DS	+0.25DS/–0.75 DC × 180	20/25	20/25
P9	52/F	–1.00DS/–1.00 DC × 45	–0.75 DS/–1.00 DC × 165	20/20	20/20
P10	27/F	+0.25 DS/–0.75 DC × 155	Plano/–0.75 DC × 5	20/20	20/20
P11	67/F	+3.00 DS/–0.75 DC × 22	+1.75 DS/–0.50 DC × 159	20/20	20/25

BCVA, best-corrected visual acuity; F, female; M, male; DS, diopter sphere; DC, diopter cylinder.

a At first study visit.

b Cystoid macular edema.

c Intraocular lens.

### Testing With the Modified Humphrey Field Analyzer

**Figure 1.**
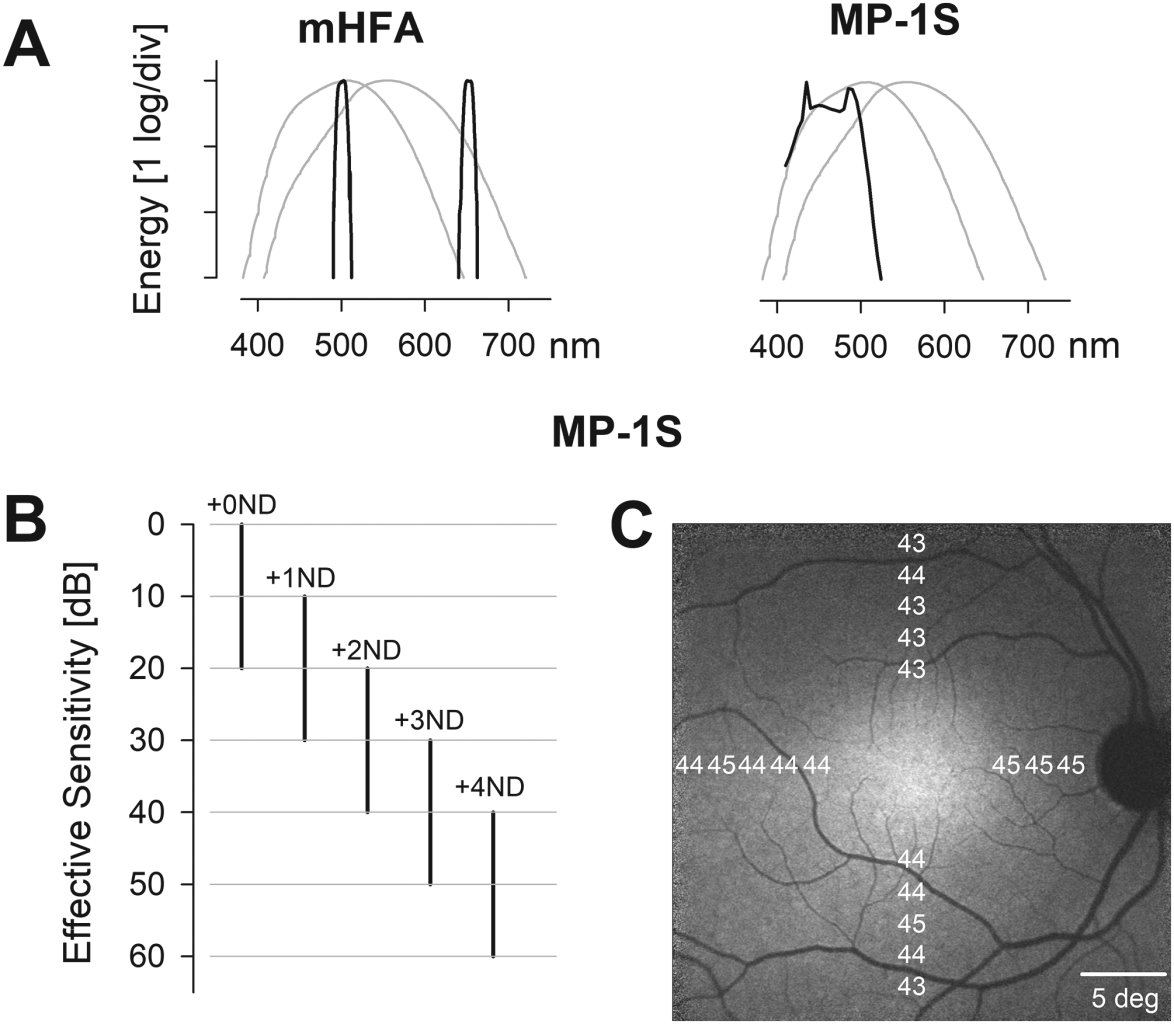
Evaluating macular rod function using the mHFA and MP-1S. **(A)** Spectral distribution of the monochromatic blue and red colored stimuli used with the mHFA (*left*) and the broadband blue stimulus used with the MP-1S (*right*), superimposed over the human scotopic and photopic luminosity functions (*thin gray curves*). **(B)** Effective sensitivities obtained with the MP-1S by extending the limited dynamic range of the blue colored stimuli with the use of a range of ND filters. Each log unit of ND filter (+1 to +4 ND) shifts the instrument dynamic range to lower luminances by 10 dB. **(C)** A total of 18 locations were sampled at 2° intervals, between 6° and 14° eccentricity from the fovea along the horizontal and vertical meridia. The mean effective thresholds for the MP-1S in controls did not vary substantially across the test locations.

Testing of dark-adapted RP eyes was performed with the modified Humphrey field analyzer (mHFA), as previously described.^19^^,^^20^ We considered the mHFA to be the gold standard, as it has been extensively applied to developing our understanding of a wide range of IRDs and investigations of potential treatments over the last 35 years.^21^^–^^30^ The technique of dark-adapted chromatic perimetry (DACP) was used with the mHFA. DACP uses two colors to define the sensitivity and photoreceptor type mediating the perception.^19^^,^^20^^,^^31^^–^^34^ The two colors used with the mHFA were blue (500 nm) and red (650 nm) (Fig. 1A, left) generated with narrow-band interference filters; stimuli had 1.73° diameter (Goldmann size V), 200-ms duration, and a 50-decibel (dB) instrumental dynamic range.

### Testing With the Scotopic Microperimeter

The retina-tracking microperimeter MP-1S (Nidek Technologies, Padova, Italy) is a modified “scotopic” version of the standard MP-1 instrument that includes a filter holder in the stimulus path.^11^^,^^12^ Spectra and luminance of white stimuli are shaped by the introduction of different combinations of color and neutral-density (ND) filters. Macular function was assessed using broadband shorter wavelength stimuli (Fig. 1A, right) produced with a blue transmitting filter (52-532; Edmund Optics, Barrington, NJ). The MP-1S has an instrumental dynamic range of 20 dB (2 log units), which was shifted to dimmer stimulus luminances with the addition of ND filters ranging from 1 to 4 log units with 1-log steps (65-817, 65-820, 65-822, 36-276; Edmund Optics), in addition to the color filter (Fig. 1B). Stimulus conditions used for the MP-1S are referred to here as the color plus ND filter; for example, B+1 refers to the combination of a blue filter and a 1 ND filter, and B+0 refers to a blue filter without a ND filter. Stimuli had 1.73° diameter (Goldmann size V) and 200-ms duration; the background selected was red (which was also filtered through the blue filter before reaching the subject's eye). The subjects were shielded from stray light originating from the instrument or the computer screen.

### Test Locations and Analysis

Both instruments sampled locations along the horizontal and vertical meridia between 6° and 14° eccentricity at 2° intervals (Fig. 1C). The lower limit of eccentricity was chosen to avoid the effects of macular pigment, and the upper limit was constrained mostly by the field of view. Photoreceptor mediation at each locus was determined by mHFA sensitivities.^19^^,^^20^ Only locations showing rod mediation with blue mHFA stimuli were retained; cone-mediated locations were censored. The sensitivity loss (SL) for mHFA testing was calculated at each locus from the previously published normal values (*n* = 20).^20^ For MP-1S testing, effective sensitivities at each locus were calculated first by adding the ND value to the raw sensitivity value. SL for the MP-1S was then calculated as a difference between average effective sensitivity of control subjects for each locus and the corresponding effective sensitivity for subjects with RP. Left eye results were transformed to equivalent right eye coordinates. A transition zone locus was defined on mHFA results as the most eccentric test location with a SL of 30 dB or less that was followed by another more eccentric location that demonstrated at least 10-dB greater loss. Test loci identified as transition zone were grouped together and analyzed separately from the remaining non–transition-zone test loci. Near-infrared excited reduced-illuminance autofluorescence imaging (NIR-RAFI) was done as previously described.^35^ Agreement between the mHFA and MP-1S was assessed by the 95% confidence interval (CI) for the paired difference in SLs obtained from both instruments. The interval was estimated as 1.96 × SD, where SD is the total standard deviation from a mixed-effects model with subject and eye (nested) random effects to account for internal correlation of the data.^36^ Similarly, the test–retest variability was quantified using the coefficient of repeatability (CR), calculated as 1.96 × SD, where SD is the total standard deviation from a mixed-effects model for the difference in SL between the two study visits. Confidence intervals for upper and lower limits and for the means were obtained by bootstrapping (*n* = 500) over subjects. Analyses were performed using the lme4 (version 1.1-27) package from R 3.6.3 (R Foundation for Statistical Computing, Vienna, Austria).

## Results

### Finding the Appropriate Dynamic Range in Controls

The limited instrument dynamic range of the MP-1S necessitates attenuation of the stimuli to allow for dark-adapted testing in controls. Previously, a combination of blue and 2 ND filters (referred to as B+2 in the current work) was used to measure normal dark-adapted sensitivities.^11^ Subsequently, combinations of blue and 0, 1, or 2 ND filters were used in normal subjects and patients.^12^^–^^14^^,^^18^^,^^37^ There was evidence of a ceiling effect in normal subjects at the perifoveal locations with the B+2 filters.^11^^,^^12^^,^^14^ The ceiling effect implied that the instrument was not producing dim enough stimuli to measure true dark-adapted sensitivities. We performed preliminary experiments with the B+2 combination and found a ceiling effect in at least 14 of 18 test locations. The B+3 and B+4 conditions, however, appeared to avoid both floor and ceiling effects in control eyes. Consequently, we assessed controls with the B+4 condition to minimize the effective background level as much as possible.

### Macular Rod Function in Controls and RP Patients

In controls, the average effective MP-1S sensitivities with blue stimuli were relatively homogeneous across the horizontal and vertical meridia (average ± SD, 43.5 ± 1.0 dB; range, 43–45 dB) (Fig. 1C), providing a potential dynamic range for estimating SL of more than 40 dB. The equivalent mHFA sensitivities with blue stimuli at the same locations were also nearly homogeneous (49.8 ± 0.86 dB; range, 48–51 dB; not shown) and provided a potential dynamic range of nearly 50 dB for estimating SL.

**Figure 2.**
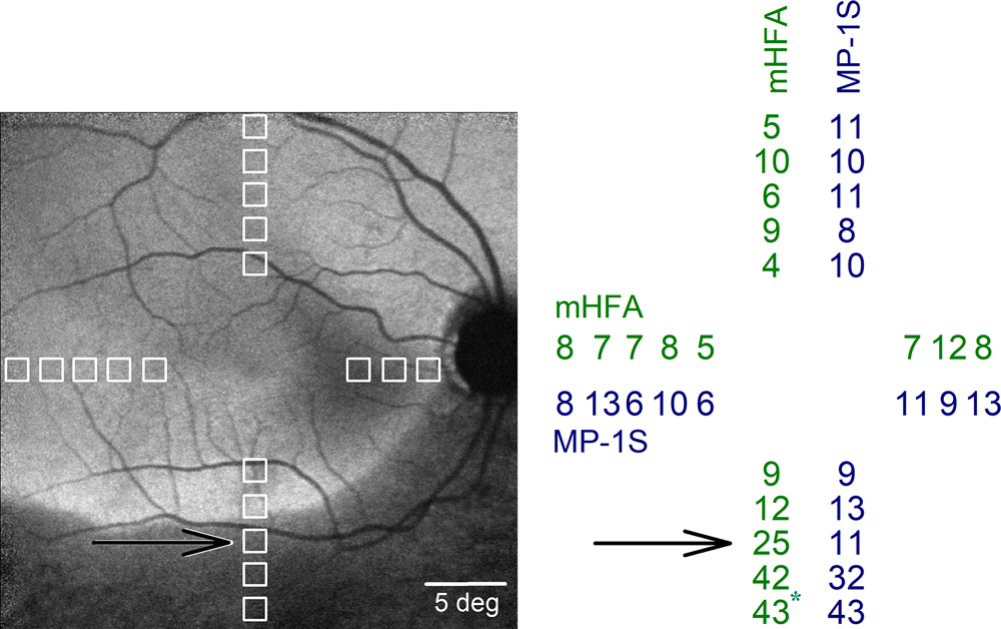
Residual macular rod function in a representative subject with retinitis pigmentosa. The 18 macular test locations (*left*, *white squares*) spanning the central 28° are shown superimposed on the near-infrared excited reduced-illuminance autofluorescence image. Regions with retained retinal pigment epithelium (RPE) melanization appear brighter than regions with RPE demelanization, which are relatively darker (inferior retina). The test location near the transition between these two regions is indicated (*black arrow*). The sensitivity loss (SL in dB, *right*), estimated as the difference between average effective threshold of control subjects for each locus and the corresponding threshold in this subject, was similar for the two perimeters for most locations except at the transition zone (*black arrow*). ^*^Cone-mediated location.

In patients with RP, most of the sensitivities were lower than controls, and appropriate filter combinations were used to avoid floor and ceiling effects with the MP-1S. Results from a representative patient with RP illustrate an example comparing the macular rod function estimated by the MP-1S to that estimated by the mHFA (Fig. 2). NIR-RAFI^35^ provides a qualitative distribution of disease across the macula (Fig. 2, left). In this imaging modality, areas with retained RPE melanization are depicted with higher NIR-RAFI intensity, whereas areas with RPE demelanization secondary to retinal degeneration correspond to lower intensity. There is an arcuate boundary in the inferior macula showing a distinct transition from health to disease. Perimetric test locations along the vertical and horizontal meridia are shown (Fig. 2, left, white squares). All of the superior, nasal, and temporal retinal samples and two of the inferior retinal samples fall within the healthier retinal regions, whereas three inferior retinal samples fall within the area of apparently greater disease.

With the mHFA, all of the locations within the healthier regions showed mild SL values smaller than 12 dB (Fig. 2, right, green). For the MP-1S, B+3 and B+0 conditions were used to obtain microperimetric sensitivities avoiding floor and ceiling values, and SL values were estimated (Fig. 2, right, blue). In general, there was close correspondence between SL estimates of the two perimeters. The main exception was the location identified as the transition zone using mHFA criteria (Fig. 2, black arrow). The difference between the estimates of SL by the two perimeters across 17 non-transition zone locations was 1.24 ± 4.07 dB, whereas at the transition zone the difference was 14 dB.

Across all 22 RP eyes, 396 test locations were assessed with both perimeters. After censoring locations with cone mediation (*n* = 34) and those with a SL of greater than 30 dB and the loci with no sensitivity (*n* = 81), 281 locations were identified as rod mediated by the mHFA and blue stimuli. Of these selected locations with mild to moderate rod-mediated SL, 259 were identified as being in non-transition zones and 22 in transition zones. Among test locations identified as non-transition zone, 67, 169, 5, and 18 were assessed using B+3, B+2, B+1, and B+0 filters, respectively. SL estimates with the two perimeters were 9.5 (±2.4) versus 6.2 (±2.0) dB for B+3, 12.7 (±4.9) versus 10.1 (±4.3) dB for B+2, and 19.4 (±2.9) versus 17.2 (±5.3) dB for B+1, respectively, for the MP-1S and the mHFA. The instruments showed the largest difference for B+0, with SL of 31.2 (±3.9) versus 25.2 (±3.7) dB for the MP-1S and the mHFA, respectively.

**Figure 3.**
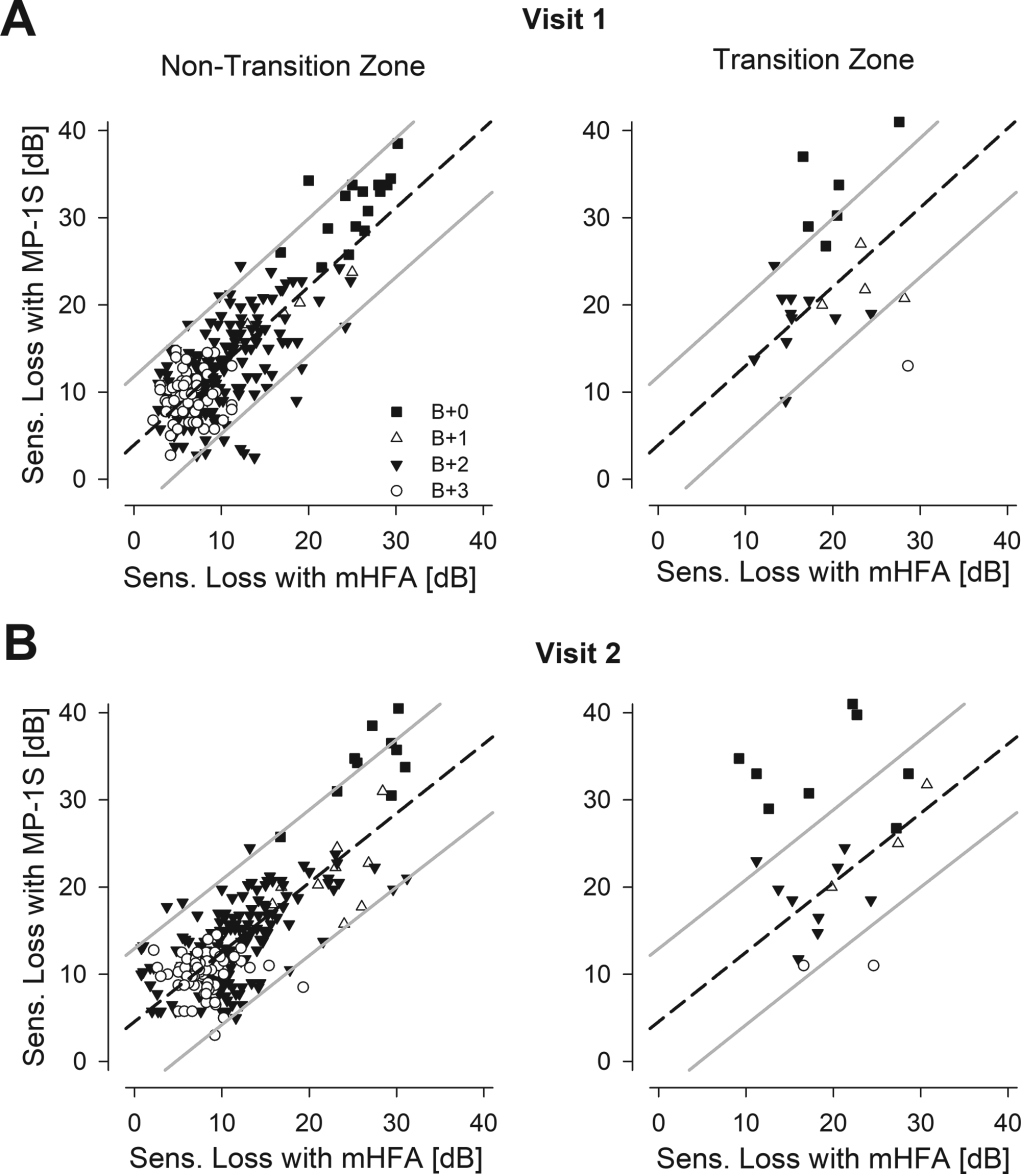
Comparison of the SL estimated from the two perimeters in all eyes with retinitis pigmentosa. **(A)** The estimated MP-1S SL for the majority of the samples not located at a transition zone (left, *n* = 259) were highly correlated with the corresponding mHFA SL. Different symbols (+0 ND, *filled square*; +1 ND, *unfilled triangle*; +2 ND, *filled triangle*; +3 ND, *unfilled circle*) represent the range of ND filters used for the MP-1S testing. The relationship among the SL estimates was well fit by a linear regression (dashed line). The 95% prediction intervals are also shown (*gray lines*). The regression fit and prediction interval from the non–transition-zone data were used to understand the relationship at limited transition zone loci (right, *n* = 22). The SL estimates at the transition zones (*right*) tended to show greater mismatches between the two perimeters. **(B)** Results from a second study visit displayed comparably to those shown in panel A.

The SL estimates using the MP-1S in non-transition zones could be simply predicted from a linear function of SL estimates using the mHFA (Fig. 3A, left). The regression coefficient was high (*r* = 0.80; CI, 0.75–0.84), but there was a small offset and a non-unity slope (MP-1S = 0.9 × mHFA + 3.9 [dB]). The relationship between the two perimeters was more complex at the transition zone. Nearly half of the transition zone loci showed a substantial mismatch between the SL estimates originating from the two perimeters (Fig. 3A, right). At a second visit 6 months later, the relationship between the two perimeters was like that of the first visit (*r* = 0.76; CI, 0.71–0.81) (Fig. 3B). In terms of agreement, the mean difference (bias) between instruments (MP-1S – mHFA) was 2.7 dB (CI, 1.6–3.6). The 95% limits of agreement spans were −5.5 dB (CI, −7 to −3.6) and 10.9 (CI, 9.7–12.1). There was no substantial dependence of the difference or its variance with mean SL level (0.08 and 0.05 dB/dB slopes for difference and absolute residuals, respectively).

### Test–Retest Variability

**Figure 4.**
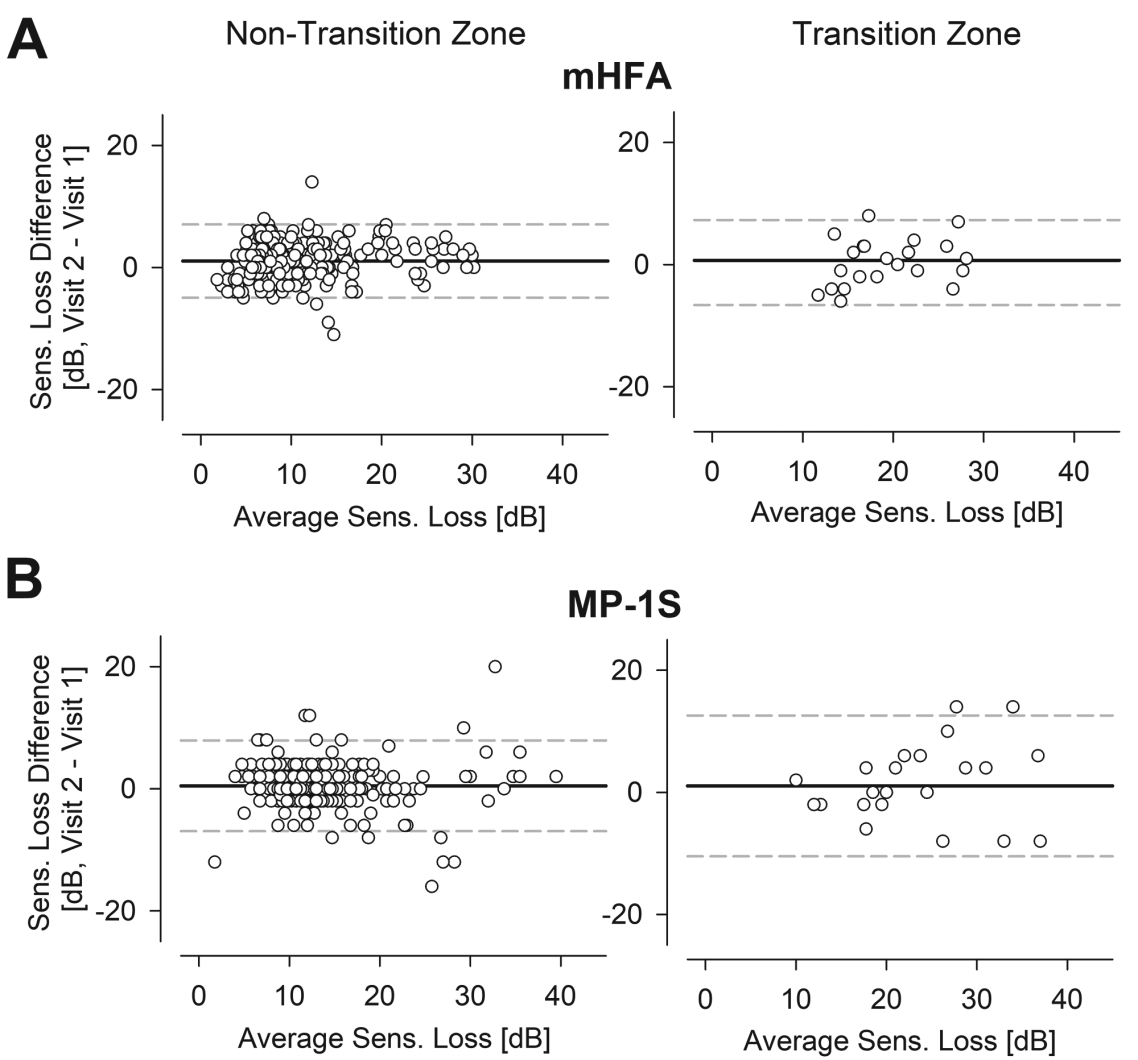
Test–retest variability of the SL estimated from the two perimeters with blue stimuli in the non–transition-zone and transition-zone loci in subjects with RP. Bland–Altman plots revealed some variability across two consecutive tests 6 months apart for the mHFA **(A)** and the MP-1S **(B)**, with higher variability for loci in the transition zone (*right*) as opposed to the loci in the non-transition zones (*left*).

Next, we explored the test–retest variability of macular rod function by evaluating the CR across two visits. Bland–Altman plots summarizing pointwise SL differences for the mHFA at non-transition zones showed no evidence of a systematic relationship between variability and mean sensitivity (Fig. 4A, left); the CR was 5.9 dB, with a 95% interval for differences of −4.7 dB (CI, −5.8 to −3.4) and 7.1 dB (CI, 6.3–7.6). For the MP-1S, there was some suggestion of greater variability with greater SL values (Fig. 4B, left); the CR was 8.2 dB, with a 95% interval for differences of −7.6 dB (CI, −11.1 to −5.24) and 8.3 dB (CI, 6.3–10.5). As anticipated, the results for both the MP-1S and mHFA were more variable at the transition zones (Figs. 4A, 4B, right panels). The CR values at the transition zones were 7.5 dB and 12.5 dB for the mHFA and MP-1S, respectively. Small biases of 1.2 and 0.4 dB for the mHFA and MP-1S, respectively, could potentially reflect evidence of disease progression during the 6-month interval.

## Discussion

The aim of the current work was to estimate the validity and reliability of macular rod sensitivity measured with the MP-1S by comparing it to the mHFA, as a ground truth is difficult to establish for a psychophysical measure. We demonstrated that the results of the MP-1S microperimeter were highly correlated with those for the mHFA; however, the MP-1S tended to show somewhat larger variability and tended to overestimate the rod sensitivity loss especially at retinal loci with greater disease.

Common features of all RP caused by mutations in many different genes are dysfunction and degeneration of retinal photoreceptors. In many cases, rod photoreceptors are affected earlier or more severely than cone photoreceptors,^27^^,^^38^ but there are also examples of variegated distribution of rod and cone disease across retinas.^28^ Sampling of visual function across the retina can provide estimates of underlying photoreceptor disease. Standard perimeters are used to obtain cone function,^3^^,^^29^^,^^39^^–^^42^ but assessment of rod function topography across the retina tends to be more challenging.^43^ The DACP technique has been used to identify and map rod function, and there are several perimeters that can perform DACP.^12^^,^^17^^,^^19^^,^^20^^,^^42^^–^^49^ Underlying technological platforms vary substantially among the perimeters. It would be ideal to attain platform-independent measurement of DACP, but there are no standards for measuring exceedingly dim stimulus lights associated with dark-adapted thresholds or for evaluating effective background light experienced by the subject due to incomplete blocking of extraneous light sources. An alternative approach would be to quantitatively compare results obtained with different perimetric platforms. We are not aware of previous cross-platform investigations of rod function in retinal disease.

For the current work, we compared the mHFA to the MP-1S. The former is a free-viewing perimeter where stimuli are projected inside a white bowl. The latter is a retina-tracking perimeter where the stimuli are displayed on a liquid-crystal display screen coupled to the eye in Maxwellian view. We considered the mHFA to be the gold standard, as it has contributed to the largest published collection of DACP results in patients with IRDs over the last 40 years.^4^^–^^6^^,^^10^^,^^19^^–^^24^^,^^26^^–^^30^^,^^42^^,^^47^^–^^56^ We directly compared dark-adapted rod function measured with the MP-1S scotopic microperimeter to that measured with the mHFA in patients with different disease stages. We limited the comparison to overlapping capabilities of both platforms: macular localization of stimuli and stably fixating subjects.

Measurement of macular rod function has intrinsic scientific value. At intermediate and severe stages of disease in many IRDs, the only remaining rod function can often be in the macula.^30^ In addition, most subretinal gene therapies tend to treat the macular region.^57^ Reliable localization of residual macular rod function and distinguishing it from residual cone function will be critical in following up patients with IRDs who partake in observational and interventional clinical trials.

An important assumption implicit in all free-viewing perimeters is stable and foveal fixation whereas retina-tracking platforms can compensate for fixation abnormalities. When evaluating mid- and far-peripheral retina, fixation challenges tend to have little consequence, and free-viewing perimetric platforms such as mHFA can still be used.^58^ But, when evaluating macular function, small changes in fixation can have major consequences in retinal localization of function.^10^^,^^16^^–^^18^^,^^59^ In the current work we argued that cross-platform comparability must be demonstrated first in stably fixating patients before the reliability of the MP-1S for estimating macular rod function in patients with abnormal fixation can be considered.

In addition, many patients with IRDs have annular transition zones where there are steep changes from vision to scotoma corresponding structurally to changes from retained retina to degeneration. Retinal disease tends to progress faster near transition zones.^60^^,^^61^ Previous work has shown that free-viewing perimeters have high variability near scotoma boundaries,^62^^,^^63^ whereas retina-tracking perimeters (microperimeters) can avoid this increased variability in some cases^10^^,^^61^ but not in others.^64^^,^^65^ The current work focused on macular areas of relatively homogeneous function in patients with good fixation to fairly compare a free-viewing perimeter with a microperimeter. There were also preliminary results in transition zones, but future studies would have to expand on this important subject area.

At locations preselected to retain rod-mediated function, there was a close linear relationship between SLs estimated with the mHFA and those with the MP-1S over a 3-log unit range of disease severities, and this relationship held similarly on a second visit 6 months later. However, SLs derived from the MP-1S were on average about 3 dB larger than SLs derived from the mHFA. One of the possible reasons for the discrepancy in SL could be the results from the control subjects. Ideally, a large number of control subjects should have been evaluated with both perimeters, but this was not within the scope of the current work. Instead, we used previously published normal results for the mHFA^20^ and evaluated a small number of control subjects with the MP-1S. Another reason for the mismatch could be the use of ND filters to expand the dynamic range of the MP-1S. All control subjects were measured with a 4 ND filter, whereas patients were evaluated with 0 to 3 ND filters. The use of smaller ND filters could have reduced sensitivities, as greater amounts of the dim background light of MP-1S would be transmitted to the subject. Consistent with this hypothesis, the largest difference between the mHFA and MP-1S results occurred when no ND filter was used.

Dark-adapted sensitivity to blue stimuli is not always mediated by rod photoreceptors. Use of red stimuli allows determination of the rod-versus-cone origin of the measurement, and this is the premise of the DACP, which was used with the mHFA results in the current work. Others have previously used the DACP method with the MP-1S and have claimed to distinguish between rod and cone mediation.^12^^,^^15^ We chose not to study this approach in the MP-1S based on preliminary experiments that showed a 1.3-log unit reduction of sensitivity in normal eyes for the red stimuli when comparing results with the 0 ND to those of the 2 ND filter. We interpreted our preliminary results to mean that the dim red background of the MP-1S filtered through the red filter was illuminating the retina of the subjects at high enough luminances to cause substantial reduction of dark-adapted sensitivity.^7^ Blue stimuli of the MP-1S were likely not as affected, due to the blocking of the red background by the blue filter. Future studies will be required to better understand the filtering of background light and its impact on dark-adapted sensitivity.

In conclusion, the close correlation between the results for the MP-1S and the mHFA, as well as the repeatability of this relationship at a second visit, suggest that the scotopic microperimeter can be used in interventional trials or in natural history studies to evaluate rod disease progression. However, the MP-1S is less likely to provide a good estimate of the absolute rod sensitivity loss necessary for estimating treatment potential in quantitative comparisons of structure versus function.^30^
